# Reduction of Thermal Conductivity for Icosahedral Al-Cu-Fe Quasicrystal through Heavy Element Substitution

**DOI:** 10.3390/ma14185238

**Published:** 2021-09-12

**Authors:** Yoshiki Takagiwa, Ryota Maeda, Satoshi Ohhashi, An-Pang Tsai

**Affiliations:** 1 National Institute for Materials Science (NIMS), 1-2-1 Sengen, Tsukuba 305-0047, Ibaraki, Japan; ryota-maeda@denka.co.jp; 2 Institute of Multidisciplinary Research for Advanced Materials (IMRAM), Tohoku University, 2-1-1 Katahira, Sendai 980-8577, Japan; satoshi.ohhashi.d4@tohoku.ac.jp (S.O.); aptsai@tagen.tohoku.co.jp (A.-P.T.)

**Keywords:** quasicrystals, thermal conductivity, Al-Cu-Fe, heavy element substitution, thermoelectric properties

## Abstract

Icosahedral Al-Cu-Fe quasicrystal (QC) shows moderate electrical conductivity and low thermal conductivity, and both p- and n-type conduction can be controlled by tuning the sample composition, making it potentially suited for thermoelectric materials. In this work, we investigated the effect of introducing chemical disorder through heavy element substitution on the thermal conductivity of Al-Cu-Fe QC. We substituted Au and Pt elements for Cu up to 3 at% in a composition of Al_63_Cu_25_Fe_12_, i.e., Al_63_Cu_25−*x*_(Au,Pt)*_x_*Fe_12_ (*x* = 0, 1, 2, 3). The substitutions of Au and Pt for Cu reduced the phonon thermal conductivity at 300 K (*κ*_ph,300K_) by up to 17%. The reduction of *κ*_ph,300K_ is attributed to a decrease in the specific heat and phonon relaxation time through heavy element substitution. We found that increasing the Pt content reduced the specific heat at high temperatures, which may be caused by the locked state of phasons. The observed glass-like low values of *κ*_ph,300K_ (0.9–1.1 W m^−1^ K^−^^1^ at 300 K) for Al_63_Cu_25−*x*_(Au,Pt)*_x_*Fe_12_ are close to the lower limit calculated using the Cahill model.

## 1. Introduction

Thermoelectric materials can directly convert a temperature difference into electrical voltage. Home heating, automotive exhaust, and industrial processes all generate enormous waste heat. Thermoelectric materials can recover waste heat emitted from commercial and industrial cycles. The potential of thermoelectric materials can be evaluated by the dimensionless figure of merit *zT*, as expressed by

(1) $$zT=\frac{S^{2}\sigma }{\kappa_{t}}T,$$

where *S*, *σ*, *κ*_t_, and *T* are the Seebeck coefficient, the electrical conductivity, the total thermal conductivity, and the temperature, respectively [1,2]. In general, *κ*_t_ is the sum of two contributions, the phonon part *κ*_ph_ and the electron part *κ*_el_.

(2) $$\kappa_{t}=\kappa_{\mathrm{ph}}+\kappa_{\mathrm{el}}$$

To enhance *zT*, it is necessary to simultaneously optimize *S*, *σ*, and *κ*_t_. Icosahedral quasicrystals (QCs) have shown good thermoelectric properties because of the formation of the pseudogap and complex crystal structures [3]. We succeeded in an enhancement of *zT* for Al_71_Pd_20_Mn_9_ QC from 0.18 to 0.26 through 3 at% Ga substitution for Al without reducing the power factor *S*^2^*σ*. Substitution of Ga at less than 4 at% for Al had less influence on both *σ* and *S*, while *κ*_t_ (in particular, *κ*_ph_) decreased through the combination of weakening of the intercluster bonds and an alloying effect [3,4,5].

Icosahedral Al-Cu-Fe QC shows the following attractive characteristics as a potential thermoelectric material:

(i) The magnitude and sign of the Seebeck coefficient strongly depend on the sample composition, i.e., the position of the Fermi level in the electronic density of states, indicating that both p- and n-type materials can be obtained in the same alloy [6,7,8].
(ii) As expected from a complex crystal structure, a low *κ*_t_ of less than 2 W m^−1^ K^−1^ at 300 K has been reported [7,8,9,10].
(iii) The constituent elements are nontoxic, readily available, and show favorable costs for industrial use [11].

Although several advantageous physical properties are recognized in Al-Cu-Fe QC, there are only a few studies on the high-temperature thermoelectric properties [9,12]. Until now, the effect of elemental substitution on the thermoelectric properties of Al-Cu-Fe QC has not been clarified. In particular, lowering *κ*_ph_ using an alloying effect, inspired from previous works [4,13,14] on Al-Pd-Mn and Al-Pd-Re QCs by transition metal substitutions, remains a possibility. We also expected that the suppression of phason flipping by heavy element substitution could reduce the thermal conductivity. In this work, we focus on icosahedral Al-Cu-Fe QC for lowering *κ*_t_ and investigate the effects of Au and Pt substitutions for Cu on *κ*_ph_. Here, we selected Au and Pt as substitution elements for Cu because of the large atomic radii, strong bond strength with Al, and a high melting point of Pt. From the experimental point of view, investigating high-temperature thermal conductivity is of great importance for controlling the unusual increase in the specific heat [15,16,17,18,19] for Al-based QCs and approximants, which provides an additional route to tune the thermoelectric [3] and thermal rectifier [12] properties.

## 2. Experimental Procedure and Sample Characterization

**Figure 1:**
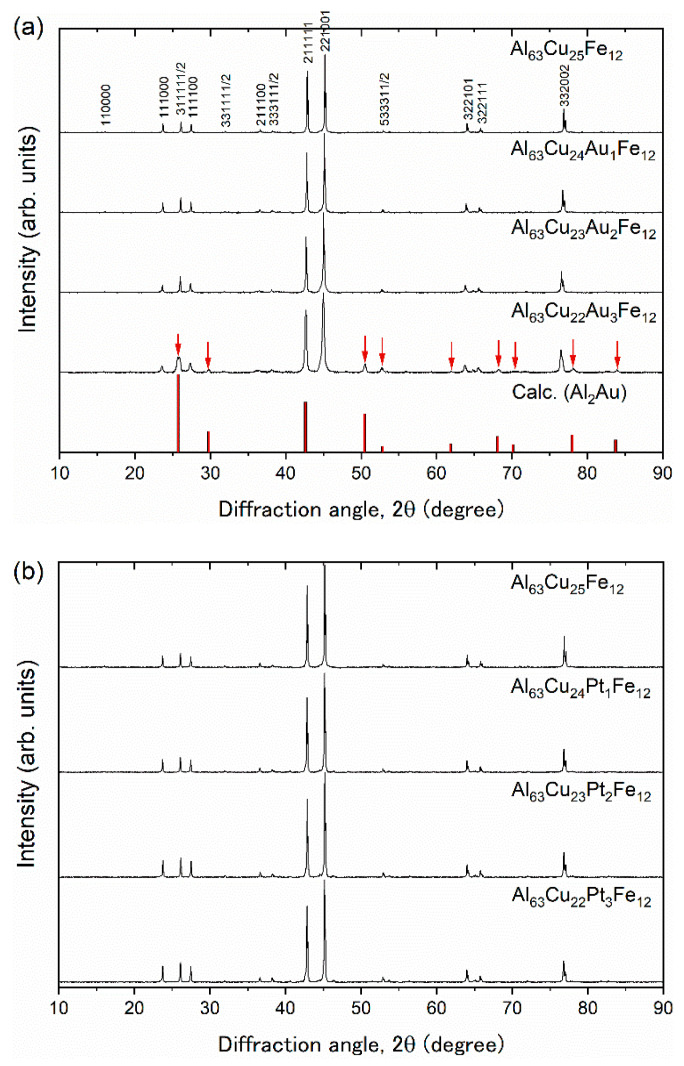
X-ray diffraction patterns of (**a**) Al_63_Cu_25−*x*_(Au,Pt)*_x_*Fe_12_ (*x* = 0, 1, 2, 3), together with peak indices and calculated peak positions of Al_2_Au, and (**b**) Al_63_Cu_25−*x*_Pt*_x_*Fe_12_ (*x* = 0, 1, 2, 3). Arrows indicate peaks of excess phase of Al_2_Au.

**Figure 2:**
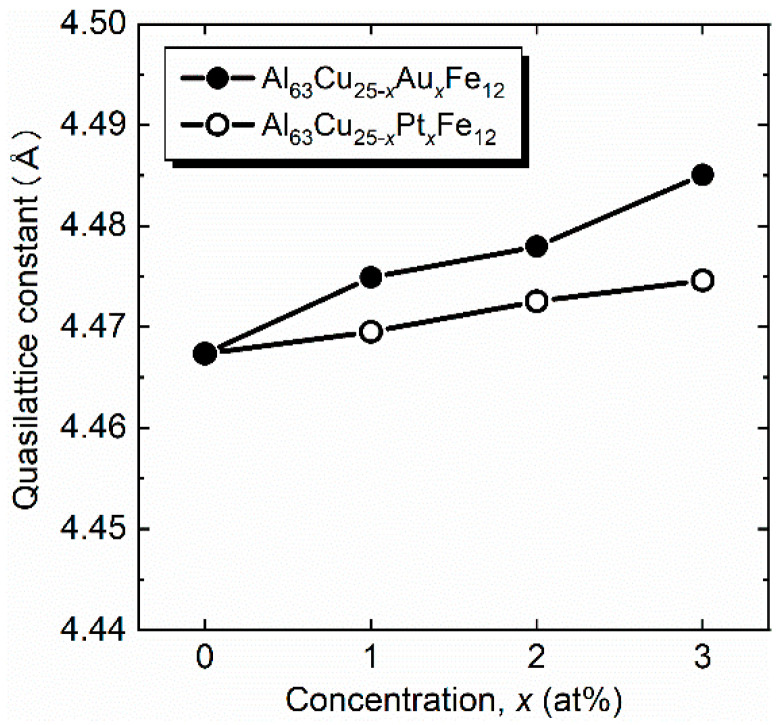
Quasilattice constant of Al_63_Cu_25−*x*_(Au,Pt)*_x_*Fe_12_ (*x* = 0, 1, 2, 3).

Mother ingots of Al_63_Cu_25−*x*_(Au,Pt)*_x_*Fe_12_ (*x* = 0, 1, 2, 3) were synthesized by an arc melting technique and annealed at 1073 K for 48 h under a purified argon atmosphere. The obtained ingots were crushed to a particle size of less than 45 µm. The regulated powder samples were put into a carbon die with an inner diameter of 10 mm for spark plasma sintering with a heating rate of ~150 K min^−1^ (LABOX-110MC; SinterLand, Inc., Niigata, Japan). A pressure of 57 MPa was applied under a purified argon atmosphere during the sintering process. The consolidating temperature was maintained for 10 min for all samples. Table 1 lists applied consolidating temperatures, bulk densities obtained from geometric calculations, and crystalline sizes using Scherrer’s formula for Al_63_Cu_25−*x*_(Au,Pt)*_x_*Fe_12_ (*x* = 0, 1, 2, 3). The resulting relative densities were over 95%. The bulk density increased with increasing Au and Pt fraction *x*, which can be understood as increasing the average mass through heavy element substitution. The estimated crystalline sizes were slightly larger than the value (850 Å) of the NIST Si standard powder sample. The phase purity of the samples was evaluated by X-ray diffraction (XRD) (SmartLab; Rigaku, Inc., Tokyo, Japan), as shown in Figure 1a,b. We observed peak shifting to a lower degree with increasing (Au,Pt) concentrations, indicating that the quasilattice constant increased (Figure 2). This behavior can be qualitatively explained by the substitution of larger atomic radii of Au (1.37Å) and Pt (1.39Å) for Cu (1.28Å). Only the sample of Al_63_Cu_22_Au_3_Fe_12_ contained a small amount of the excess phase of Al_2_Au. The precipitates can affect the thermoelectric properties; thus, we excluded the results and discussion for Al_63_Cu_22_Au_3_Fe_12_. Composition analyses were performed using inductively coupled plasma atomic emission spectroscopy (ICP) analysis (Table 2). We found that the analyzed Au/Pt concentration increased with increasing nominal fraction, except for the sample of Al_63_Cu_22_Au_3_Fe_12_, in which the secondary phase of Al_2_Au was precipitated. From ICP analysis, we found that Au hardly substitutes for Cu, while Pt can substitute for Cu up to 3 at%.

The total thermal conductivity *κ*_t_ was calculated from the density *d*, the specific heat at constant pressure *C*_P_, and the thermal diffusivity λ using the relationship *κ*_t_ = *d*∙*C*_P_∙*λ*. Both *C*_P_ and *λ* were measured by the laser flash method (TC-7000; Advance Riko, Inc., Kanagawa, Japan) from 300 to 873 K. The transverse and longitudinal speeds of sound were measured by ultrasonic pulse-echo method (Echometer 1062; Nihon Matech Corp., Tokyo, Japan). The electrical conductivity was measured between 300 K and 873 K by the four-probe method (ZEM-3; Advance Riko, Inc., Kanagawa, Japan) for a rough estimation of the electron thermal conductivity *κ*_el_ using the Wiedemann–Franz law. The Seebeck coefficient was obtained by the steady-state temperature gradient method using a ZEM-3 instrument.

## 3. Results and Discussion

**Figure 3:**
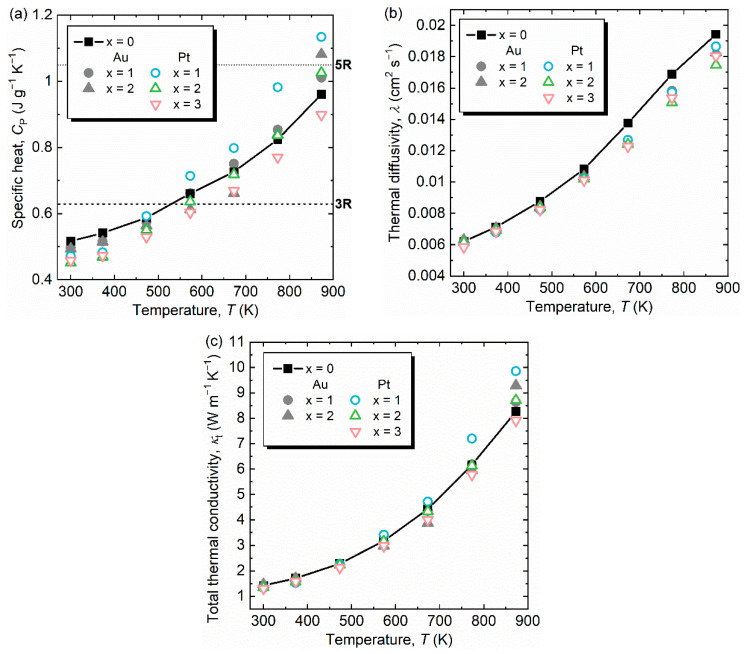
(**a**) Specific heat at constant pressure *C*_P_, (**b**) thermal diffusivity *λ*, and (**c**) total thermal conductivity *κ*_t_ as a function of temperature for Al_63_Cu_25−*x*_(Au,Pt)*_x_*Fe_12_ (*x* = 0, 1, 2, 3). The dashed and dotted lines represent the Dulong–Petit limit (3*R*) and 5*R*, respectively. Al_63_Cu_22_Au_3_Fe_12_ is excluded because of secondary phase precipitation.

Figure 3a,b show the temperature dependences of *C*_P_ and *λ* from 300 to 873 K for Al_63_Cu_25−*x*_(Au,Pt)*_x_*Fe_12_ (*x* = 0, 1, 2, 3). The measured *C*_P_ of Al_63_Cu_25_Fe_12_ increased with increasing temperature and reached between 4*R* and 5*R* at 873 K, as shown in Figure 3a. This behavior is quantitatively consistent with a previous report by Prekul et al. [16]. An unusual increase in the *C*_P_ of icosahedral and decagonal QCs was discussed in terms of the introduction of chemical disorder and anharmonicity in lattice vibration [15] and the localized electronic nature [16] of QCs. Edagawa et al. first mentioned that excess specific heat can be attributed to the excitation of phasons [15], which will be discussed below. The values of *C*_P_ at 300 K for both Au- and Pt-substituted samples decreased with increasing *x* because of an increase in mean atomic weight. However, the trend of *C*_P_ at high temperatures is rather complex; the *C*_P_ values of Au- and Pt-substituted samples, except for a sample with *x* = 3 (Pt), were larger than that of pristine Al-Cu-Fe. Here, we exclude the detailed discussion on *C*_P_ of Au-substituted samples because Au did not systematically substitute for Cu, as already mentioned in Section 2. It is easily expected that increasing Pt fraction will bring chemical disorder in the atomic arrangement. Therefore, the observed increase in *C*_P_ at high temperatures for samples with *x* = 1 and 2 can be caused by introducing chemical disorder. On the contrary, the systematic decrease in *C*_P_ at 873 K with increasing Pt fraction was observed; in particular, the *C*_P_ of the sample with *x* = 3 was lower than that of Al_63_Cu_25_Fe_12_ QC. The observed reduction of *C*_P_ at high temperatures may be attributed to the locked state of phasons, that is, a pinning effect of phasons through heavy element substitution for Cu sites. However, there is room for further discussion on the effect of excited or locked phasons on the high-temperature specific heat, which will enhance our knowledge of the specific features of QCs. On the other hand, as expected, the *λ* values were reduced by both Au and Pt substitutions throughout the measurement temperature region because of the alloying effect, as shown in Figure 3b.

The temperature dependence of obtained *κ*_t_ for all samples is displayed in Figure 3c. The value of *κ*_t_ at 300 K (*κ*_t,300K_) for Al_63_Cu_25_Fe_12_ is 1.42 W m^−1^ K^−1^; previously reported *κ*_t,300K_ of Al-Cu-Fe QC is distributed in the range of 1−2 W m^−1^ K^−1^ [7,8,9,10], which is caused by the difference in the sample composition because *κ*_t_ (in particular, *κ*_el_) is sensitive to the actual composition, as observed in Al-Pd-Re QC [20]. However, the details are not clear at this stage because the analyzed composition and each parameter of *d*, *C*_P_, and *λ* were not described in the literature [7,8,9,10]. Compared with other Al-based QCs, the measured *κ*_t,300K_ of Al_63_Cu_25_Fe_12_ is significantly higher than those of Al-Pd-Mn and Al-Pd-Re QCs [3,20] because of the relatively light constituent elements Fe and Co, compared with Pd and Re. A detailed comparison will be discussed below.

**Figure 4:**
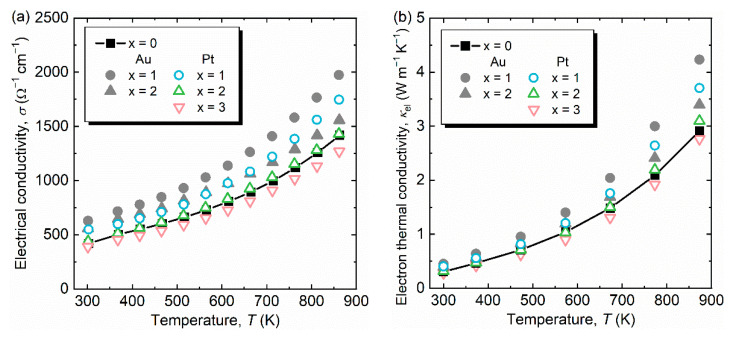
(**a**) Electrical conductivity *σ* and (**b**) electron thermal conductivity *κ*_el_ as a function of temperature for Al_63_Cu_25−*x*_(Au,Pt)*_x_*Fe_12_ (*x* = 0, 1, 2, 3). Al_63_Cu_22_Au_3_Fe_12_ is excluded because of secondary phase precipitation.

Table 3 lists the *κ*_t,300K_ for all samples investigated. While the *κ*_t,300K_ for Au-substituted samples increased by up to 2.8%, the *κ*_t,300K_ for Pt-substituted samples decreased by up to 7.7%. To understand the different behaviors of *κ*_t,300K_ with Au and Pt substitutions, we evaluated the lattice component of *κ*_ph_ after subtracting *κ*_el_ from *κ*_t_ using the Wiedemann–Franz law:

(3) $$\kappa_{\mathrm{ph}}=\kappa_{t}-\kappa_{\mathrm{el}}=\kappa_{t}-L_{0}\sigma T$$

where *L*_0_ is the Lorenz number. We estimated *L*_0_ value using a model proposed by Kim et al., *L*_0_ = 1.5 + exp[−|*S*|/116]*10^−8^ V^2^ K^−2^ [21]. The electrical conductivity *σ* and calculated *κ*_el_ as a function of temperature are displayed in Figure 4a,b. All samples show semiconductor-like behavior, i.e., σ increases with increasing temperature [Figure 4a]; thus, the estimated *κ*_el_ also increases monotonically [Figure 4b]. The measured *σ* at 300 K (*σ*_300K_) for Al_63_Cu_25_Fe_12_ was approximately 400 Ω^−1^ cm^−1^, which is consistent with previous data of similar sample compositions of Al-Cu-Fe QC [6,7,8,10]. Contrary to expectation, *σ* significantly increased with Au substitution (*σ*_300K_ ~700 Ω^−1^ cm^−1^), although Cu and Au have the same number of electrons per atom ratio (e/a) of +1 [22]. Referring to the results of ICP composition analyses (Table 2), the Al concentration of Au-substituted samples increased as *x* increases. Therefore, the measured enhancement of *σ* will be caused by the different Al/(Cu+Au) ratios, i.e., shifting the position of the Fermi level in the electronic density of states. On the other hand, Pt substitution for Cu succeeds in more precise composition control with almost constant Al concentration. The sample with *x* = 1 (Pt) possessed a higher *σ*_300K_ of 550 Ω^−1^ cm^−1^, probably because of an increase in the carrier concentration, compared with the sample with x = 0. In turn, the carrier mobility of samples with higher Pt fractions of *x* = 2, 3 will be largely suppressed by introducing chemical disorder, resulting in a decrease in σ for the samples with *x* = 2 and 3. Although the microstructure (such as defect, strain, and grain size) can also affect *σ*, the observed non-monotonic change in *σ* cannot be explained only by such extrinsic factors.

Returning to the estimation of *κ*_el_, the Wiedemann–Franz law applied for pseudogap and narrow-gap compounds is found to be invalid because it assumes that the spectral conductivity varies linearly with energy [17]. The validity of the Wiedemann–Franz law was also discussed by Maciá [23]. Hitherto, there is no empirical relation to calculate *κ*_el_ for QCs; thus, we adopted the conventional Equation (3) and *L*_0_ values using an empirical model [21] for a rough estimation of *κ*_ph_.

**Figure 5:**
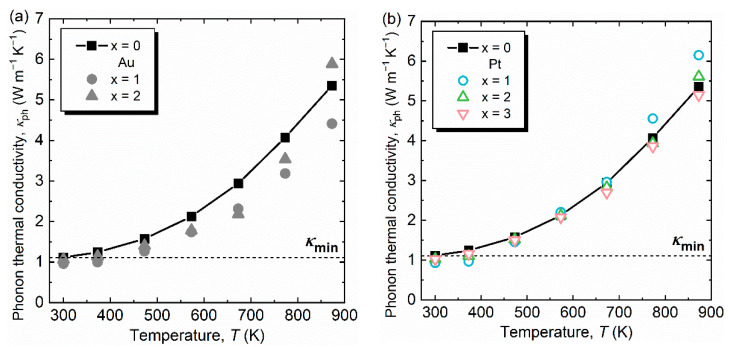
Phonon thermal conductivity *κ*_ph_ as a function of temperature for (**a**) Al_63_Cu_25−*x*_Au*_x_*Fe_12_ (*x* = 0, 1, 2) and (**b**) Al_63_Cu_25−*x*_Pt*_x_*Fe_12_ (*x* = 0, 1, 2, 3). The dashed lines represent the minimum thermal conductivity *κ*_min_ for Al_63_Cu_25_Fe_12_.

The calculated *κ*_ph_ as a function of temperature are shown in Figure 5a,b. It should be noted that although the apparent increase in *κ*_ph_ at high temperatures originates from conduction carriers [17], room-temperature *κ*_ph_ may not be largely under- or over-estimated. We found that the increase in *κ*_t,300K_ for Au-substituted samples (Table 3) is attributed to an increase in *κ*_el_, as shown in Figure 4b, probably because of an increase in the carrier concentration because the electrical conductivity increases for Au-substituted Al-Cu-Fe [Figure 4a]. The *κ*_ph_ at 300 K (*κ*_ph,300K_) for both Au- and Pt-substituted samples decreased by up to 14.3% and 17.0%, respectively (Table 3), which is caused by the alloying effect. For a better understanding of the decrease in *κ*_ph,300K_, we performed speed-of-sound measurements for pristine and Au- and Pt-substituted samples. The obtained speeds of sound *v*_s_ are distributed between 4150–4400 m s^−1^, and no composition dependence of *v*_s_ is observed. Here, *κ*_ph_ is expressed using the specific heat at constant volume *C*_V_, *v*_s_, and the phonon relaxation time *τ*_ph_,

(4) $$\kappa_{\mathrm{ph}}=\frac{1}{3}C_{V}v_{s}^{2}\tau_{\mathrm{ph}}$$

We now compare each parameter change of *C*_P_ and *v*_s_, as listed in Table 3. Here, we should discuss *C*_V_ rather than *C*_P_ using the following relationship [15]:

(5) $$C_{V}=C_{P}-9VB\alpha^{2}T$$

where *V*, *B*, and *α* are the atomic volume, the bulk modulus, and the linear thermal expansion coefficient, respectively. Although we did not perform measurements of *B* and *α*, qualitative analysis can be performed using the parameter *C*_P_. The rate of change in *C*_P_, *ΔC*_P,300K_/*C*_P,300K_, increased with increasing Au/Pt fraction; the reduction of *C*_P_ at 300 K for Pt-substituted samples is larger than that for Au-substituted samples. On the other hand, the rate of change in *v*_s_, *Δv*_s_/*v*_s_ had less influence on *κ*_ph,300K_. However, the additional reduction of *τ*_ph_ should be considered to explain *Δκ*_ph,300K_/*κ*_ph,300K_ of 14.3% and 17.0% for Au- and Pt-substituted samples, respectively. The reduction of *τ*_ph_ is estimated to be up to ~11%, and the alloying effect through heavy element substitution, in particular, worked in dilute Au- and Pt-substituted Al-Cu-Fe QC.

Next, we evaluated the minimum thermal conductivity *κ*_min_ using the model proposed by Cahill et al. [24,25], which provides the lower limit of the lattice thermal conductivity for amorphous solids and disordered crystals. The *κ*_min_ can be calculated as the following equation,

(6) $$\kappa_{\min}={\left(\frac{\pi }{6}\right)}^{\frac{1}{3}}k_{B}n^{\frac{2}{3}}\underset{l,t}{\sum }v_{l,t}{\left(\frac{T}{\theta_{l,t}}\right)}^{2}\int_{0}^{\frac{\theta_{l,t}}{T}}\frac{x^{3}e^{x}}{{\left(e^{x}-1\right)}^{2}}dx$$

Here, *k*_B_ is the Boltzmann constant, *n* is the number density of atoms, *v*_l_ is the longitudinal speed of sound, *v*_t_ is the transverse speed of sound, *T* is the temperature, and *θ*_l,t_ is the cut off temperature, $\theta_{l,t}=v_{l,t}\left(\frac{\hbar }{k_{B}}\right){\left(6\pi^{2}N\right)}^{\frac{1}{3}}$, where ℏ is Planck’s constant. The calculated *κ*_min_ is listed in Table 3. Note here that the values of *κ*_ph,300K_ for Al_63_Cu_25−*x*_(Au,Pt)*_x_*Fe_12_ are already close to the *κ*_min_, indicating that extra phonon engineering such as nanostructuring [26] will not be beneficial for further reduction of *κ*_ph_ of the present materials.

**Figure 6:**
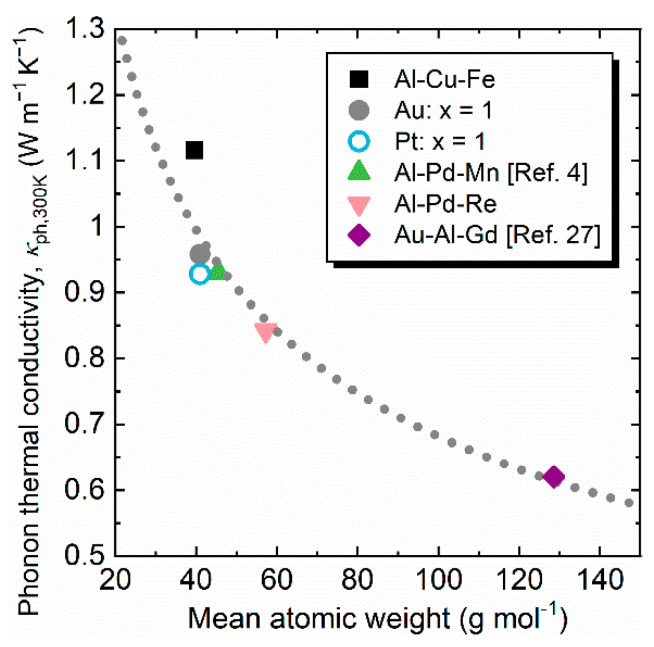
Phonon thermal conductivity at 300 K *κ*_ph,300K_ versus mean atomic weight for various undoped icosahedral quasicrystals (QCs) and related approximant crystals (ACs): Al-Cu-Fe QC, Al-Cu-(Au,Pt)-Fe QCs, Al-Pd-Mn QC [4], Al-Pd-Re QC, and cubic-Au-Al-Gd AC [27]. The Au/Pt substitution reduced *κ*_ph,300K_ down to that of Al-Pd-Mn QC [4]. The dashed curve is drawn to guide the eye.

Finally, we compare *κ*_ph,300K_ for various three-dimensional icosahedral QCs and related approximant crystals (AC): Al-Cu-Fe QC, Al-Cu-(Au,Pt)-Fe QCs, Al-Pd-Mn QC [3], Al-Pd-Re QC, and Tsai-type cubic-Au-Al-Gd AC [27], as shown in Figure 6. Compared with Al-based ternary QCs, Al-Cu-Fe QC having a lighter mean atomic weight shows the highest *κ*_ph,300K_, while Al-Pd-Re QC shows the lowest one. A significant increase in *d* results in a low *v*_s_, $v_{s}=\sqrt{B/d}$. Indeed, the values of *v*_s_ are 4340, 3770, and 3590 m s^−1^ for Al-Cu-Fe QC, Al-Pd-Mn QC [4], and Al-Pd-Re QC, respectively. Note here again that the decrease in *κ*_ph,300K_ of Au- and Pt-substituted Al-Cu-Fe QCs is attributed to the reduction of both *C*_P_ and *τ*_ph_. Recently discovered Tsai-type Au-based approximant ACs have rather heavy atomic weight, close to 130 g mol^−1^. One example is cubic-Au-Al-RE ACs; a glass-like low *κ*_ph,300K_ of ~0.6 W m^−1^ K^−1^ was observed for Au-Al-Gd AC. Cubic-Au-Al-RE ACs may be a good starting point for enhancing thermoelectric properties because they possess wide composition ranges [28]. Compared with pristine Al-Cu-Fe, the Au/Pt substitution brings a heavier atomic weight of up to 10%. The *κ*_ph,300K_ of Au/Pt-substituted quaternary Al-Cu-Fe has a value close to that of ternary Al-Pd-Mn QC [3], in which a heavier mean atomic weight is expected.

## 4. Conclusions

We investigated the effects of Au and Pt substitutions on the thermal conductivity above 300 K for Al_63_Cu_25−*x*_(Au,Pt)*_x_*Fe_12_ (*x* = 0, 1, 2, 3). High-density Au- and Pt-substituted Al-Cu-Fe samples were successfully synthesized by the combination of arc melting and spark plasma sintering. We found that increasing the Pt content reduced the specific heat at high temperatures, which may be caused by the locked state of phasons. The substitution of Pt for Cu reduced *κ*_ph,300K_ by up to 17%. The reduction of *κ*_ph,300K_ was attributed to a decrease in the specific heat and phonon relaxation time through heavy element substitution and the alloying effect. The present results show that the substitution of Pt for Cu sites can reduce the *κ*_ph_ down to 0.93 W m^−1^ K^−1^ at 300 K, which is comparable with that of ternary Al-Pd-Mn QC with heavier mean atomic weight.

## Figures and Tables

**Table 1:** List of applied consolidating temperatures, bulk densities, crystalline sizes for Al_63_Cu_25−*x*_(Au,Pt)*_x_*Fe_12_ (*x* = 0, 1, 2, 3).

Samples	Consodidating Temperature (K)	Bulk Density (g cm^−3^)	Crystalline Size (Å)
Al_63_Cu_25_Fe_12_	898	4.43	1256(62)
Al_63_Cu_24_Au_1_Fe_12_	1013	4.58	1090(58)
Al_63_Cu_23_Au_2_Fe_12_	1018	4.70	824(70)
Al_63_Cu_22_Au_3_Fe_12_	1033	4.80	379(13)
Al_63_Cu_24_Pt_1_Fe_12_	948	4.66	1062(16)
Al_63_Cu_23_Pt_2_Fe_12_	968	4.86	979(17)
Al_63_Cu_22_Pt_3_Fe_12_	1083	4.88	974(16)

**Table 2:** Nominal compositions and ICP results for Al_63_Cu_25−*x*_(Au,Pt)*_x_*Fe_12_ (*x* = 1, 2, 3).

Nominal Compositions	Phase	ICP Analysis of Chemical Composition
Al_63_Cu_24_Au_1_Fe_12_	*i*	Al_63.1_Cu_24.2_Au_0.9_Fe_11.8_
Al_63_Cu_24_Au_2_Fe_12_	*i*	Al_64_Cu_22.4_Au_1.9_Fe_11.7_
Al_63_Cu_22_Au_3_Fe_12_	*i* + Al_2_Au	Al_65.7_Cu_21.2_Au_1.7_Fe_11.4_
Al_63_Cu_24_Pt_1_Fe_12_	*i*	Al_63.2_Cu_24.2_Pt_0.9_Fe_11.3_
Al_63_Cu_24_Pt_2_Fe_12_	*i*	Al_63.2_Cu_23.8_Pt_1.7_Fe_11.3_
Al_63_Cu_22_Pt_3_Fe_12_	*i*	Al_63.1_Cu_22.3_Pt_2.6_Fe_12_

**Table 3:** Total thermal conductivity at 300 K (*κ*_t,300K_) and its rate of change (*∆κ*_t,300K_/*κ*_t,300K_), phonon thermal conductivity at 300 K (*κ*_ph,300K_) and its rate of change (∆*κ*_ph,300K_/*κ*_ph,300K_), rate of change in specific heat at 300 K (*∆C*_P,300K_/*C*_P,300K_), rate of change in speed of sound (*Δv*_s_/*v*_s_), and minimum thermal conductivity at 300 K (*κ*_min,300K_) for Al_63_Cu_25−*x*_(Au,Pt)*_x_*Fe_12_ (*x* = 0, 1, 2, 3). Al_63_Cu_22_Au_3_Fe_12_ is excluded because of secondary phase precipitation.

**Samples**	***κ*** **_t,300K_**	***Δκ_t_*** **_,300K_** ***/κ_t_*** **_,300K_**	***κ*** **_ph,300K_**	***Δκ*** **_ph,300K_** ***/κ*** **_ph,300K_**
	**(W m^−1^ K^−1^)**	**(%)**	**(W m^−1^ K^−1^)**	**(%)**
*x* = 0	1.42	-	1.12	-
Au: *x* = 1	1.41	−0.7	0.96	−14.3
Au: *x* = 2	1.46	2.8	1.06	−5.4
Pt: *x* = 1	1.33	−6.3	0.93	−17.0
Pt: *x* = 2	1.35	−4.9	1.03	−8.0
Pt: *x* = 3	1.31	−7.7	1.03	−8.0
**Samples**	***ΔC*** **_P,_** **_300K_** ***/C*** **_P,_** **_300K_**	***Δv*** **_s_** ***/v*** **_s_**	***κ*** **_min,300K_**	
	**(%)**	**(%)**	**(W m^−1^ K^−1^)**	
*x* = 0	-	-	1.11	
Au: *x* = 1	−3.9	−0.2	1.11	
Au: *x* = 2	−4.5	−5.2	1.08	
Pt: *x* = 1	−8.7	−0.2	1.13	
Pt: *x* = 2	−13	−2.3	1.12	
Pt: *x* = 3	−11	0	1.14	

## Data Availability

The data presented in this study are available on a reasonable request from the corresponding author.
